# Tunable superoleophobicity *via* harnessing the surface chemistry of UV responsive titania coatings†

**DOI:** 10.1039/c8ra01458b

**Published:** 2018-04-10

**Authors:** Jitesh Barman, Sumit Kumar Majumder, Pritam Kumar Roy, Krishnacharya Khare

**Affiliations:** Electronic Paper Display Institute, South China Normal University, Higher Education Mega Center Guangzhou-510006 P. R. China jiteshb@m.scnu.edu.cn; Department of Physics, Indian Institute of Technology Kanpur Kanpur India-208016

## Abstract

Superoleophobic surfaces exhibiting tunable wettability are prepared by the combination of simple spray coating of Ultra Violet (UV) responsive titania nanoparticles and a low surface energy coating of a self-assembled monolayer (SAM) of 1*H*,1*H*,2*H*,2*H*-perflurodecyltrichlorosilane (PFDTS). Spray coating creates random micron-sized roughness with reentrant geometry, a necessary requirement for the superoleophobic surface, and a porous network at the nanometer size level, confirmed by the field emission scanning electron microscope (FE-SEM) images. By employing the rough surface and a low surface energy monolayer, the substrates possess superhydrophobicity with a water (*γ* = 72 mN m^−1^) contact angle of 163° and superoleophobicity with a decane (*γ* = 23 mN m^−1^) contact angle of 144°. Wettability of these surfaces is completely reversed to the superoleophilic state upon 6 h of UV irradiation. A quantitative X-ray photoelectron spectroscopy (XPS) analysis has confirmed the mechanism of decomposition of PFDTS molecules on the superoleophilic surfaces *via* interaction with the defect Ti^3+^ states of titania upon UV exposure. Furthermore, the superoleophobicity is restored to complete the transition cycle by changing the surface chemistry of the UV exposed surface *via* annealing and regrafting of the PFDTS monolayer.

## Abstract 2

Superoleophobic surface with tunable oil wettability is presented by controlling the surface chemistry *via* exploiting the photocatalytic activity of the titania nanoparticles under UV and regrafting of the same surface.

## Graphical abstract

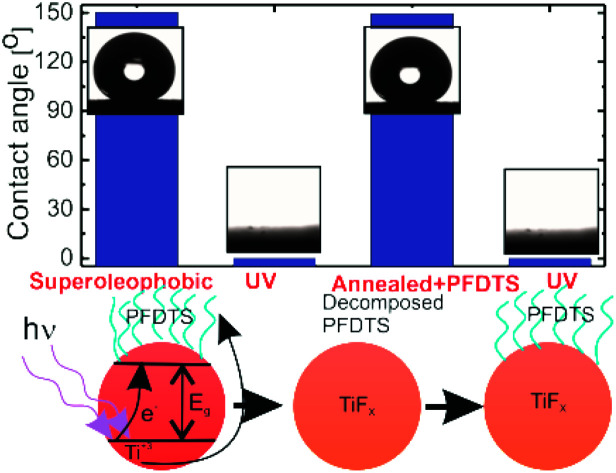

## 1 Introduction

Extreme oil repellency of a surface is a very desirable surface property for various real-life applications including the coating industry due to its plethora of interesting properties like self-cleaning^1,2^ anti-fouling,^3^ anti-smudge,^4,5^ anti-icing,^6^ self-healing^7–9^*etc.* and Lab-on-a-chip^10^ or microfluidics^11^ applications. However, achieving superoleophobicity on a surface is a challenge as the surface tension of most of the oils is below 30 mN m^−1^, that means they likely display a contact angle lower than 90° on the smooth homogeneous surface. According to the Wenzel eqn adding surface roughness on the surface will make the contact angle lower.^12^ In spite of challenges of very low Young's contact angle for oils, Tuteja *et al.* first accomplished the superoleophobicity by designing the surface roughness with special geometry called re-entrant structured roughness, which is basically an overhang shape with the wider top part and narrower bottom part close to the surface.^13^ Kim *et al.* took the surface science one step further by engineering the surface roughness with double reentrant geometry to demonstrate the superoleophobicity for liquids with zero Young's contact angle.^14^ These specially engineered surfaces can support the low surface tension liquid drops very easily by trapping more air into the space between the roughnesses under the droplet than the simple rough surface. In recent years there has been an avalanche in research studies focusing on the creation of the reentrant structured roughness using various methods like dry anisotropic etching,^15^ chemical wet etching,^16–18^ electrochemical etching,^19^ spray coating,^7,20,21^ and electrospun^22^*etc.* Noticeably, all these surfaces show constant wettability (superoleophobicity); tunable wettability for oils would offer potential promise to design and fabricate advanced materials for applications like microfluidics, oil/water separation *etc.*

Tunable wettability for oils has been achieved in an aqueous medium employing external stimuli like pH,^23–28^ electric field,^29^ thermal treatment,^30,31^ as well as light irradiation^32^*etc.* The wettability of oil inside a different liquid can be tuned either by manipulating the medium property or the surface property by external stimuli. Titania has been used extensively in the fabrication of superhydrophobic surface coatings with tunable wetting property due to its high photocatalytic activity, low cost, and in-toxicity.^33–36^ Upon exposure to the ultraviolet (UV) light, an electron from the valence band of titania makes a transition to the conduction band leaving a defect state behind which attracts hydroxyl molecules to make the substrate hydrophilic. Several studies show the switching from the superhydrophilic to the superhydrophobic state by means of dark storage^37,38^ or using post-annealing treatment.^35,36,39^ Therefore, the tuning of wetting states between hydrophobic and hydrophilic states can be achieved very easily by exposing UV light and heating treatment. Hitherto, very few studies have reported the tunable extreme oil wettability from superoleophobicity to superoleophilicity in ambient medium due to difficulty in making reentrant structured roughness on external stimuli-responsive material.^40,41^

In this article, we report a tunable superoleophobic surface based on UV responsive titania nanoparticles by harnessing the surface chemistry *via* UV exposure and annealing followed by a fresh coating of 1*H*,1*H*,2*H*,2*H*-perfluorodecyltrichlorosilane (PFDTS) monolayer. The fabricated surfaces show excellent repellency towards the water as well as low surface tension liquids like oils. Upon 6 h of UV exposure, the superoleophobic surfaces make a transition to a super wettable state displaying zero apparent contact angles towards all the liquids irrespective of their polarity. A quantitative explanation of the mechanism for transition is presented by analyzing the X-ray photoelectron spectroscopy (XPS) spectra of the superoleophobic surface before and after UV exposure. Later, the superoleophobicity was restored by annealing and regrafting a fresh layer of PFDTS molecules.

## 2 Results and discussion

### 2.1 Morphology and oil repellency of the fabricated superoleophobic surfaces

**Fig. 1:**
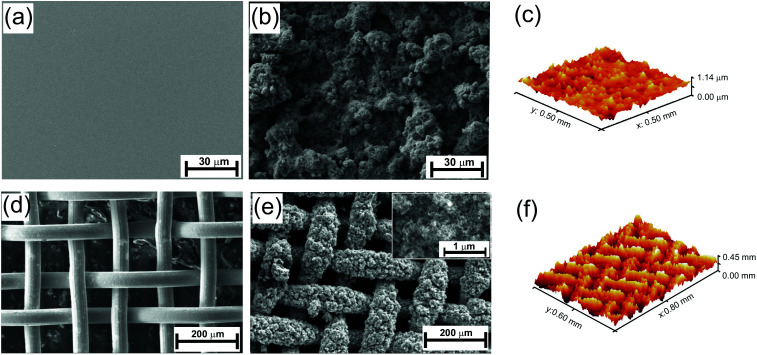
Characterization of the surface topography of spray coated Si and SS mesh substrates. (a) Pristine and (b) spray coated Si substrate. (c) 3D optical profilometry image showing roughness profile of spray coated Si substrate. FE-SEM images of (d) pristine and (e) titania coated SS mesh. Inset: magnified image of the coating showing the nanoporous topography. (f) 3D profilometry image of the titania coated SS mesh.

Fig. 1 shows the field emission scanning electron microscope (FESEM) images of the surface morphology of the TiO_2_ nano-particle coating on smooth silicon (Si) and stainless steel (SS) mesh substrates. Spray coating of nanoparticle dispersion on the substrates leads to the formation of pits or bumps with random shapes from spherical to connected cylinders with flat base structures with varying in size between 10–30 μm in lateral directions, as shown in Fig. 1b and e. The formation of the micro-bumps makes the surface very rough with RMS roughness of 0.121 μm for Si substrates and 78.8 μm for the SS mesh, estimated from the optical profilometer images as shown in Fig. 1c and f. These micro bumps were formed on a base film of TiO_2_ of thickness around 15 μm, estimated on smooth Si wafer by line scan taken on the surface perpendicular to the scratched direction (see ESI Fig. S1†). The structures possess reentrant property, broader top part compared to the base of the structure. During the time of spray coating, the particles were sprayed over the substrate which creates the microstructures from the base to top by sticking on top and side of each other leading to random shapes with special geometrical property in their structures.

The spin coating was also used to coat the dispersion but the formation of micro-bumps with reentrant structure was not observed on the surface (see ESI Fig. S2†) as the dispersion spreads over the substrate rather sprayed from the top. The effect of spray coating is more prominent on the SS mesh as the spray coated surfaces show nicely covered mesh wires with the nanoparticles as compared to the pristine one (*cf.*Fig. 1d and e). The particles cover over 70% of the open space of the SS mesh. However, the filling of the open space depends upon the spraying time which is optimised to be 2 min in our study otherwise longer or shorter spraying time would overfill even close the spacing or under-filled with particles. Inset Fig. 1e shows the magnified images of the spray coated surface exhibiting a nanoporous network of spherical nanoparticles, providing the nano level reentrant structure. In terms of the hierarchy of the structure, the image of the spray coated SS mesh shows three order of surface roughness in terms of dimension which composed of cylindrical wires (intrinsic structure of the mesh), micro bumps (due to spray coating) and nanostructure (due to intrinsic shape of the particles) all having the reentrant geometrical property.

**Fig. 2:**
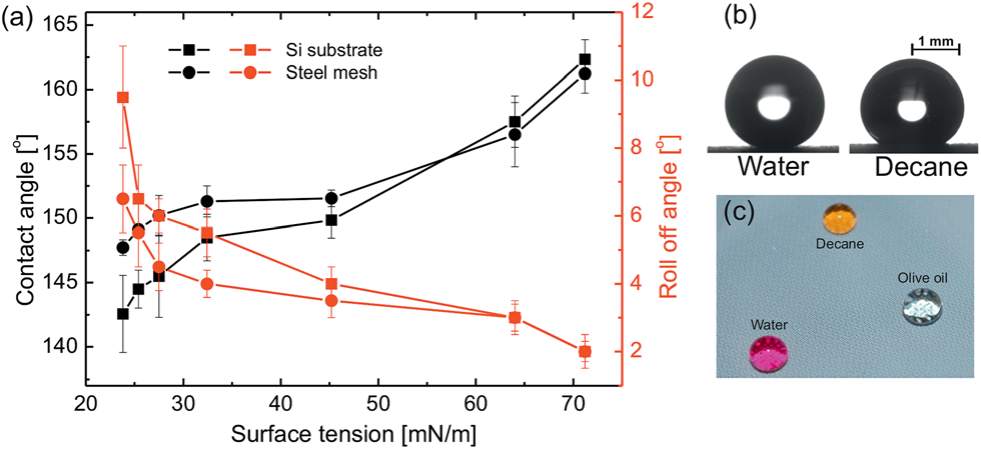
(a) Contact angle and roll-off angle as a function of different liquid surface tension on the Si and SS mesh superoleophobic surface. Optical images of the different liquids on superoleophobic (b) Si substrate and (c) SS mesh substrate.

The spray coated surfaces show superhydrophilic (superoleophilic) behavior upon depositing water drops on it due to the high surface energy of titania. The pristine homogeneous titania surface is hydrophilic in nature with an intrinsic contact angle of 50–70° for water.^42^ The apparent contact angle on the rough surface was first realised by Wenzel and the given eqn for wettability on rough surface is; cos *θ*_W_ = *r* cos *θ* where *θ* is the intrinsic contact angle of the material on smooth surface and *r* is the roughness factor.^12^ Therefore, the spreading of liquids on these surfaces with roughness was expected as the hydrophilic surfaces become more hydrophilic with addition of roughness to the surface. Moreover, the spreading state of water drop can be physically thought of imbibition of water into the micro-nano capillaries on the surface. The grafting of the low surface energy self assembled monolayer (SAM) of PFDTS molecule on the spray coated surface with very large roughness results in superhydrophobicity with very high water apparent contact angle of 165°, as shown in Fig. 2a and b. Furthermore, the extremely low roll-off angle (*α* ∼ 2°) and contact angle hysteresis (Δ*θ* ∼ 2°) authenticate the extreme repellency of these surfaces towards the water.

The superhydrophobicity of the surface can be explained by Cassie model, cos *θ*_C_ = *f*(cos *θ* + 1) − 1, which describes the apparent contact angle of water drop sitting on a rough surface with a liquid–solid area fraction of *f*.^13^ The combination of low surface energy and the hierarchical roughness created by the spray coating offers the droplet very less liquid–solid contact area fraction *via* trapping air under the droplet leads to such high superhydrophobicity. These surfaces exhibit extreme oil repellency and the low surface tension liquid like decane can easily roll off the surface (see ESI Movie S1†). The SS mesh substrate displays the contact angle 155° for glycerol, which decreases to 145° for Decane while the Si substrate shows lower repellency towards low surface tension liquids, around 141° for decane as shown in Fig. 2a. The increasing trend of roll-off angle upon decreasing the surface tension of the liquid for both the substrates confirms that the oil repellency decreases with the decrease in surface tension of the test liquids. This behavior is in line with the prediction of Cassie eqn as the apparent contact angle of the liquid decreases upon decreasing Young's contact angle (see ESI Fig. S4†), depends on the surface tension of the liquid.

**Fig. 3:**
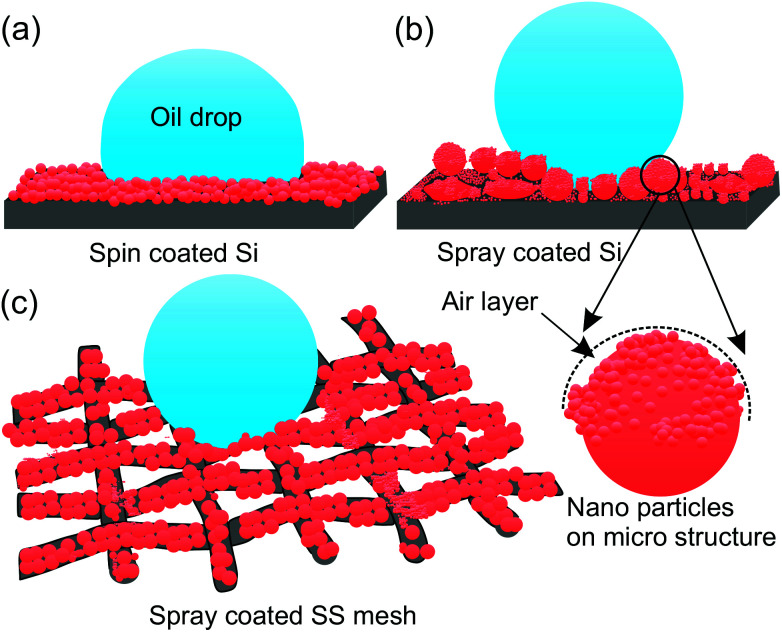
Schematic of oil drop sitting on titania substrate with (a) nano-roughness, (b) micro-nano roughness, and (c) three orders of roughness hierarchy, explaining the effect of roughness on contact angle of oils.

The higher oil repellency of SS mesh compared to the Si substrate is due to the higher surface roughness and the higher number of hierarchy in roughness dimension compared to the Si substrates which are supported by the profilometry and FE-SEM studies. The effect of the hierarchy in roughness length scale can be explained schematically in Fig. 3. According to the Cassie eqn the surface with the only nano-roughness would trap lesser amount of air under the oil droplet which results in more liquid–solid contact area fraction compared to the surface with hierarchical (micro and nano) roughness produced by the spray coating (*cf.*Fig. 3a and b). Therefore, the substrate with pristine re-entrant roughness such as SS mesh will induce higher oil repellency compared to the smooth pristine substrate.

### 2.2 Wettability conversion *via* UV irradiation and restoration of superoleophobicity

**Fig. 4:**
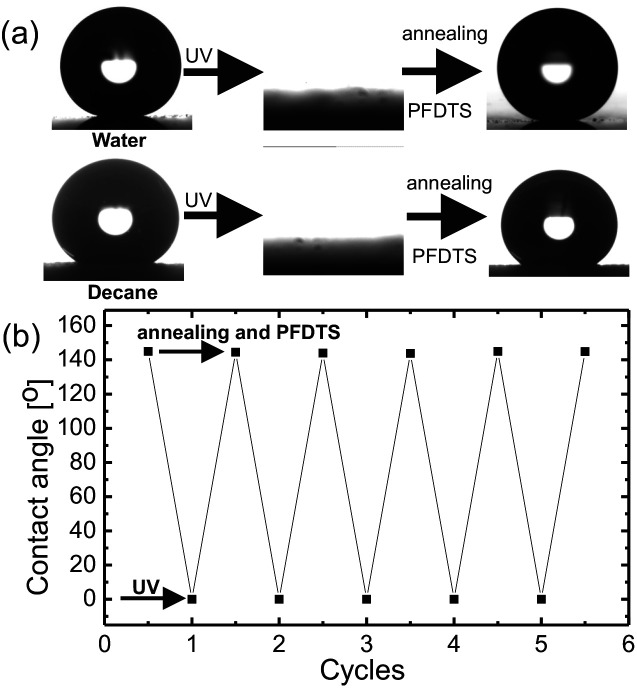
(a) Optical images showing transitions from superoleophobicity to superoleophilicity and *vice versa* upon irradiation of UV and annealing followed by re-grafting of PFDTS respectively for water and decane. (b) The contact angle of decane on the substrate at different wettability conditions for numerous cycles.

Superoleophobic surfaces reversed their wettability to superoleophilicity upon exposing to the UV of wavelength 254 nm and intensity of 365 mW cm^−2^. Decane drop spreads over the surface after 1 h of exposure and upon increasing the time of exposure to 6 h the surfaces became completely wettable for water, as shown in Fig. 4. Due to the photocatalytic activity of titania upon exposing to UV, titanium ions make a transition from the normal Ti^+4^ state to an excited state of Ti^+3^ leaving an oxygen vacancy.

**Fig. 5:**
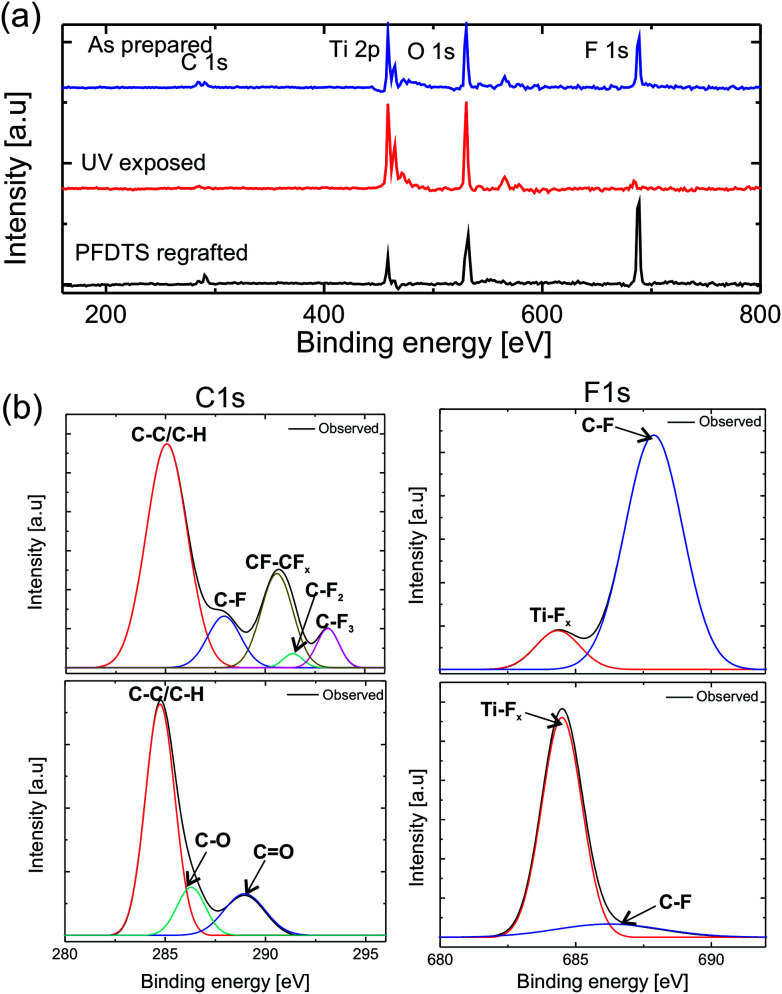
(a) XPS survey spectra showing the peaks corresponding to different elements present on the surface of as prepared superoleophobic, UV exposed and PFDTS re-grafted surface. (b) The core XPS spectra of C 1s and F 1s of the superoleophobic and UV exposed substrate.

Therefore, these oxygen vacant sites have the affinity to chemisorb polar liquid (water) molecules to show hydrophilicity. However, the photocatalytic mechanism for wettable transition doesn't fit directly in our study due to two reasons; one is that the wettability conversion was observed for both polar (water) and nonpolar (hydrocarbons and oils) liquids and there is a monolayer of PFDTS molecules on top of the TiO_2_ surface. In addition to that, we didn't observe any effect of UV irradiation on the wettability of PFDTS grafted Si smooth surface which suggests that UV doesn't affect the PFDTS molecules directly. To understand the mechanism of wettability conversion by UV on the PFDTS molecule grafted TiO_2_ surfaces, the XPS study was done before and after UV irradiation to see the change in chemical composition on the surface as shown in Fig. 5.

The XPS survey scan confirms the presence of Ti, C, O, and F elements in the superoleophobic surface whereas the UV exposed surface displays a significant decrease in peak height of F and C elements, indicates the decomposition of PFDTS molecules. Fig. 5b shows core line XPS spectra of C 1s and F 1s signals from superoleophobic and UV exposed substrates respectively. The superoleophobic substrate showed standard C bonds (C–C/C–H (285 eV)), (C–F (288 eV)), (CF–CF_*x*_ (290.6 eV)), (C–F_2_ (291.5 eV)), and (C–F_3_ (293.2 eV)) and F bonds (F–C (687.9 eV)) and (F–Ti (684.4 eV)). The UV exposed sample showed standard C bonds (C–C (285 eV), (C–O (286.1 eV), and (C=O (289.9 eV)) and F bonds (F–C (687 eV)) and (F–Ti (684.4 eV)). The binding energy values matched with the standard ref. 43–45. The evolution of new peaks in C spectrum corresponds to oxidation of carbon and the decomposition of PFDTS molecule due to UV exposure. Moreover, the increase in the intensity of the F–Ti peak in F1s spectrum after UV irradiation confirms decomposition of PFDTS molecule *via* interaction of the defect Ti^+3^ state with the PFDTS molecules. Therefore, the XPS study confirms that the chemical state of the surface was changed to hydrophilic due to UV exposure *via* the decomposition of the PFDTS molecule interacting with the defect state of Ti. The superoleophobicity of the UV exposed surfaces were restored by annealing the surfaces followed by a fresh grafting of PFDTS molecule monolayer. The switching of extreme wetting states was achieved for numerous times without any significant change in the contact angle at superoleophobic state as shown in Fig. 4b.

## 3 Experimental section

Single side polished p-type 〈100〉 silicon (Si) wafers (UniversityWafers, USA) and stainless steel (SS) meshes with 120 μm pitch (center to center) distance and 60 μm wire diameter, cut into 2 × 2 cm^2^ square pieces were used as substrates for the fabrication of superoleophobic surfaces. These substrates were thoroughly cleaned with ethanol, acetone, and toluene respectively followed by O_2_ plasma cleaning. A uniform dispersion of 6 wt% of titania nanoparticles (Sigma-Aldrich) with mean diameter 21 nm in isopropanol was prepared by ultra-sonication for 20 min. The dispersion was coated on the substrates by a spray gun following the optimized spray coating parameters; pressure and spray time as 2 kg cm^−2^ and 2 min respectively. The dispersion coated substrates were annealed at 80 °C for 30 min to evaporate the solvent. The topography of the substrates was characterized by a field-emission scanning electron microscope (FESEM, Zeiss Supra 40VP, USA). The surface roughness was estimated by analyzing the images taken by a profilometer (NanoMap (Dual mode), AEP Technology) on 1 × 1 mm^2^ area. To enhance the hydrophobicity and oleophobicity of the substrates, a self-assembled monolayer (SAM) of 1*H*,1*H*,2*H*,2*H*-perfluorodecyltrichlorosilane (PFDTS, Alfa Aesar) was grafted by vapor deposition method. The characterization of the wetting properties of the substrate was done by measuring the contact angles (Young's and apparent) by an optical contact angle Goniometer (OCA 35, Data-Physics, Germany). To avoid the deformation of all the test liquid drops due to gravity, 5 μL volume of each liquid was deposited on the substrate which yielded sessile drop with dimension sufficiently below the capillary length 
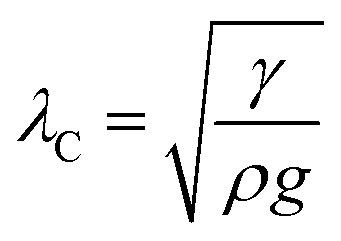
, (where *γ* and *ρ* are the surface tension, and density of the liquid respectively and *g* is the gravitational acceleration). The contact angle measurement was done when the droplet is in an equilibrium state. The reported values are the average of five measurements of contact angle at different spots on the surfaces. Deionized (DI) water (*γ* = 72 mN m^−1^), glycerol (*γ* = 64 mN m^−1^), ethylene glycol (*γ* = 47.2 mN m^−1^), olive oil (*γ* = 32 mN m^−1^), dodecane (*γ* = 25 mN m^−1^) and decane (*γ* = 23.3 mN m^−1^) were used as probe liquids to evaluate the wetting property of the substrates. The roll-off angle of 10 μL volume drop was measured by recording a movie of the rolling motion from static equilibrium while the substrate was tiled by at a tilting speed of 0.5° s^−1^ by the Goniometer, controlled by PC software (SCA 35). The percentage of various elemental compositions present on the substrates was estimated by X-ray photoelectron spectroscopy (XPS) system (PHI 5000 VersaProbe II, ULVAC-PHI Inc., Japan). The XPS analysis was carried out on the substrates for three different conditions; as prepared superoleophobic substrate, UV exposed surface, and annealed and fresh PFDTS grafted surface. The analysis of the core line spectra of the C 1s and F 1s lines was done by using deconvulate software.

## 4 Conclusions

In conclusion, the spray coating of nanoparticles is a very simple and effective method to create the hierarchical (micro and nano) reentrant surface roughnesses on various substrates, and its modification of surface chemistry by grafting a monolayer of low surface energy fluorosilane grafting can induce the superoleophobicity showing super-repellency behavior to a very low surface tension liquid such as decane. The contact angle and the roll-off angle data for SS mesh and Si substrate show that the presence of intrinsic reentrant surface roughness on the pristine substrate increases the superoleophobicity which was confirmed by the FE-SEM images of the substrates. Upon UV irradiation the superoleophobic surfaces made a transition to the super wettable state for all the liquids. The analysis of the XPS survey spectrum confirms the decomposition of PFDTS molecules on the UV exposed samples. Additionally, the wettability of PFDTS coated smooth Si substrates shows no change upon UV exposure which suggests that the decomposition of PFDTS molecules is not the directly affected by UV. The core line spectra analysis of C1s and F1s shows the formation of T–F_*x*_ chemical species on the UV exposed samples. Therefore, the UV irradiation decomposes the PFDTS molecules *via* creating a defect state in the titania due to photocatalytic action which interacts with the attached fluorosilane molecules. The superoleophobicity of the surfaces were restored by annealing and regrafting another layer of the low surface energy molecules. Therefore, the tunable extreme oil wetting states between superoleophobicity and superoleophilicity can be achieved by harnessing the chemical property of the superoleophobic surfaces.

## Conflicts of interest

There are no conflicts to declare.

## Supplementary Material

RA-008-C8RA01458B-s001

RA-008-C8RA01458B-s002
