# Supplementary material for: Tunable superoleophobicity via harnessing the surface chemistry of UV responsive titania coatings
Source: RSC Adv. 2018 Apr 10;8(24):13253–8. doi: 10.1039/c8ra01458b (PMC9079761; doi:10.1039/c8ra01458b)
Supplement: RA-008-C8RA01458B-s002 [file RA-008-C8RA01458B-s002.pdf]

Electronic Supplementary Material (ESI) for RSC Advances.
This journal is © The Royal Society of Chemistry 2018

Supplementary Information:
Tunable superoleophobicity via harnessing the surface chemistry of UV responsive titania coatings

Jitesh Barman[1,2*], Sumit Kumar Majumder[2], Pritam Kumar Roy[2], and Krishnacharya Khare[2]

[1] *Electronic Paper Display Institute, South China Normal University, Higher Education Mega Center, Guangzhou-510006, P. R. China.*
[*] *Email:jiteshb@m.scnu.edu.cn*
[2] *Department of Physics, Indian Institute of Technology Kanpur, Kanpur, India-208016.*

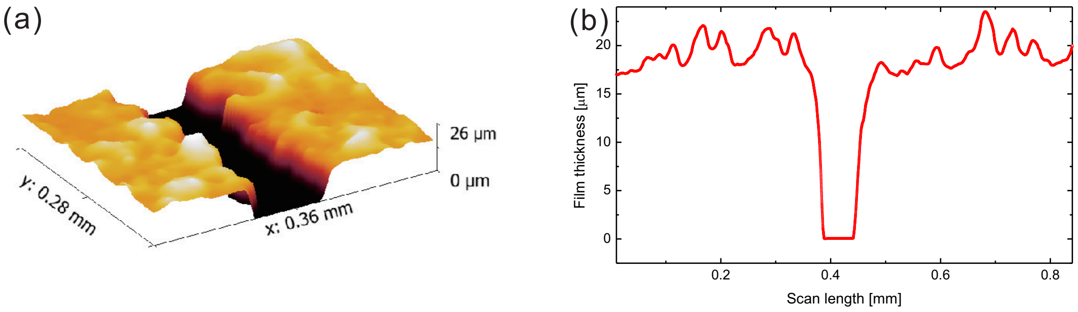

Figure S1: (a) 3D profilommtery image of the titania coated Si substrate with a scratch on the coated film (b) Line scan showing the thickness and the surface feature of titania coated Si substrate.

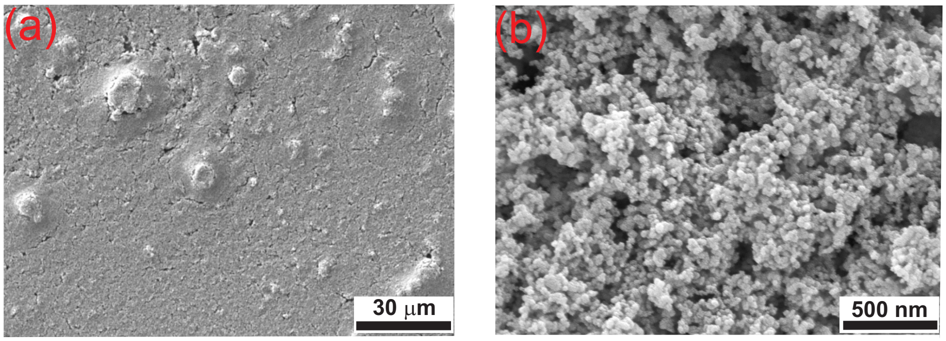

Figure S2: a) FE-SEM images of the spin coated titania surfaces showing less micron sized roughness with no reentrant property. (b) Magnified image displaying the nano porous structure of titania nanoparticles.

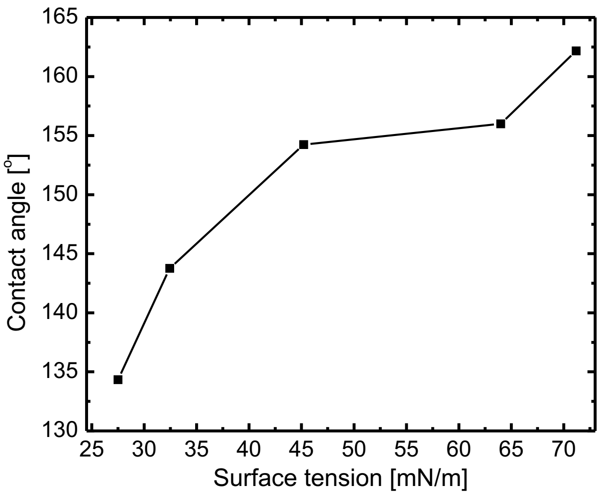

Figure S3: Apparent contact angles as function of test liquid surface tensions on oleophobic surface prepared by spin coating method.

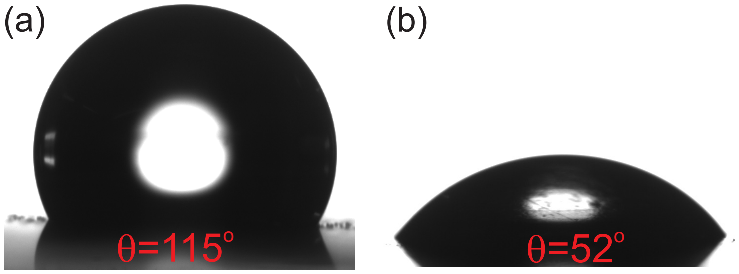

Figure S4: Young's contact angle of (a) water (= 72 mN/m) and (b) decane (= 23.3 mN/m) on PFDTS coated smooth Si substrate to show the effect of test liquid surface tension on the Young's contact angle.
